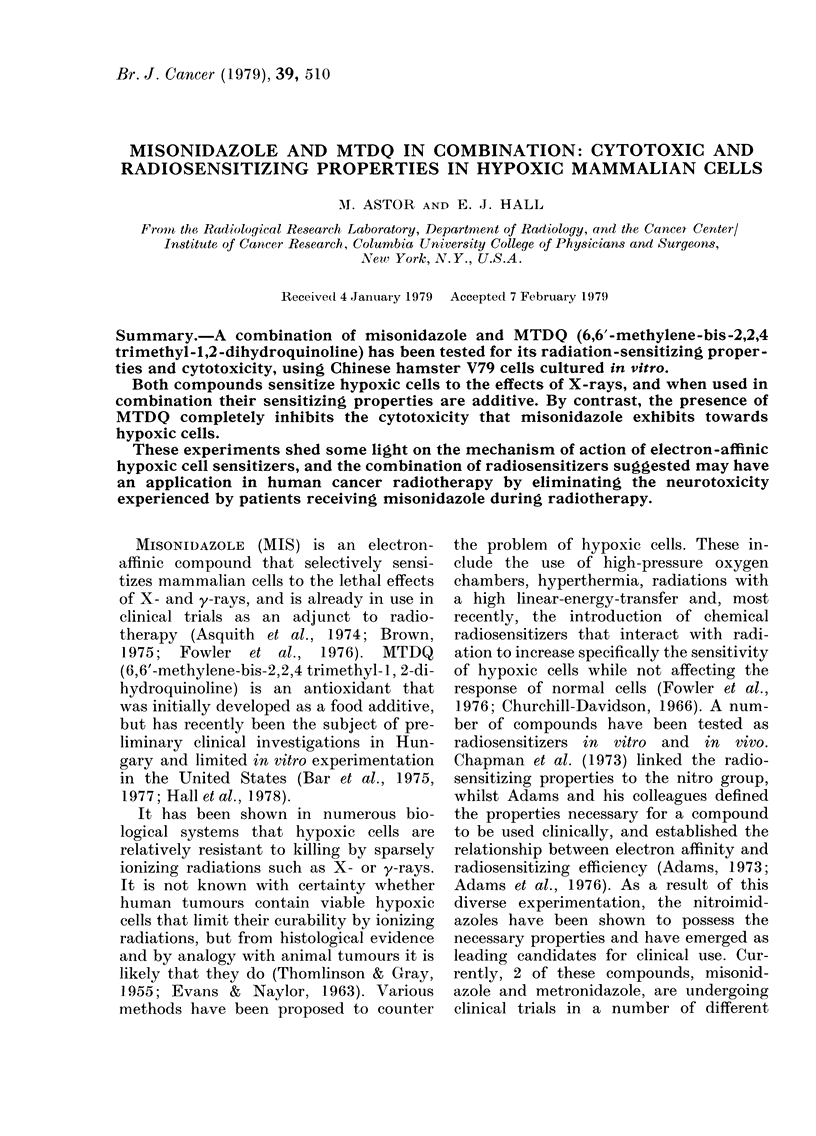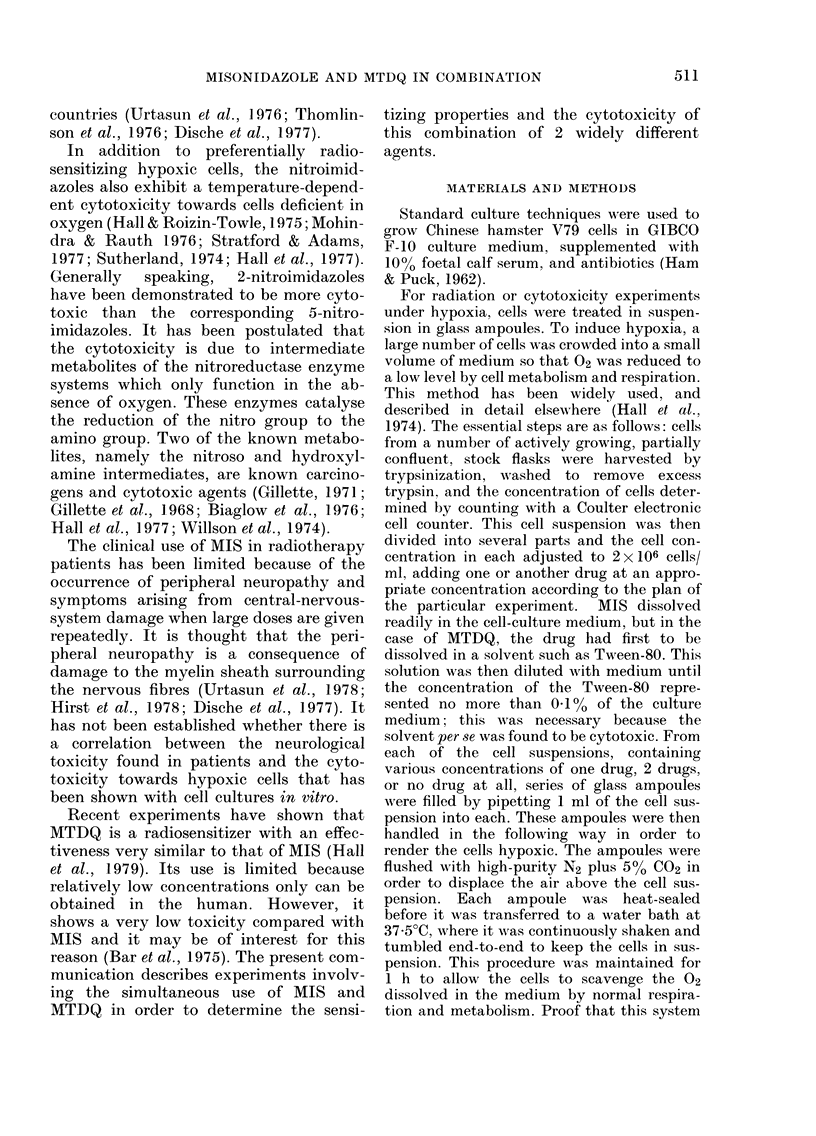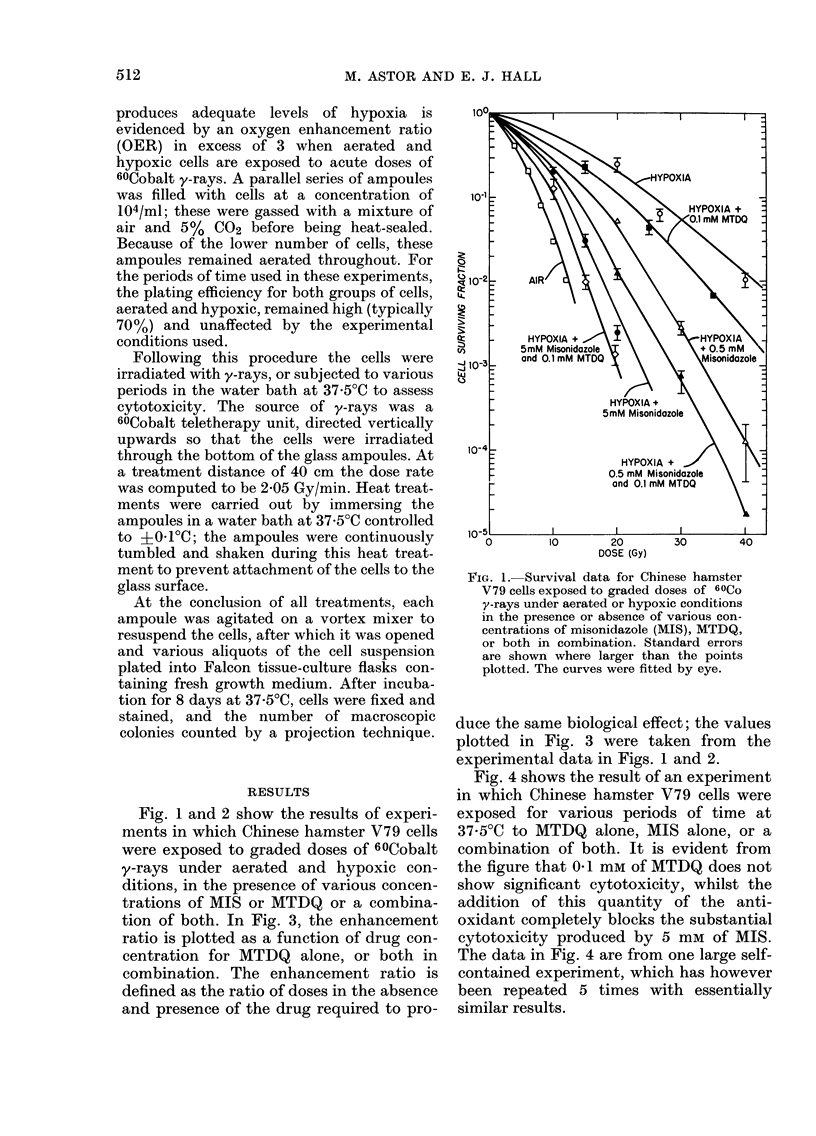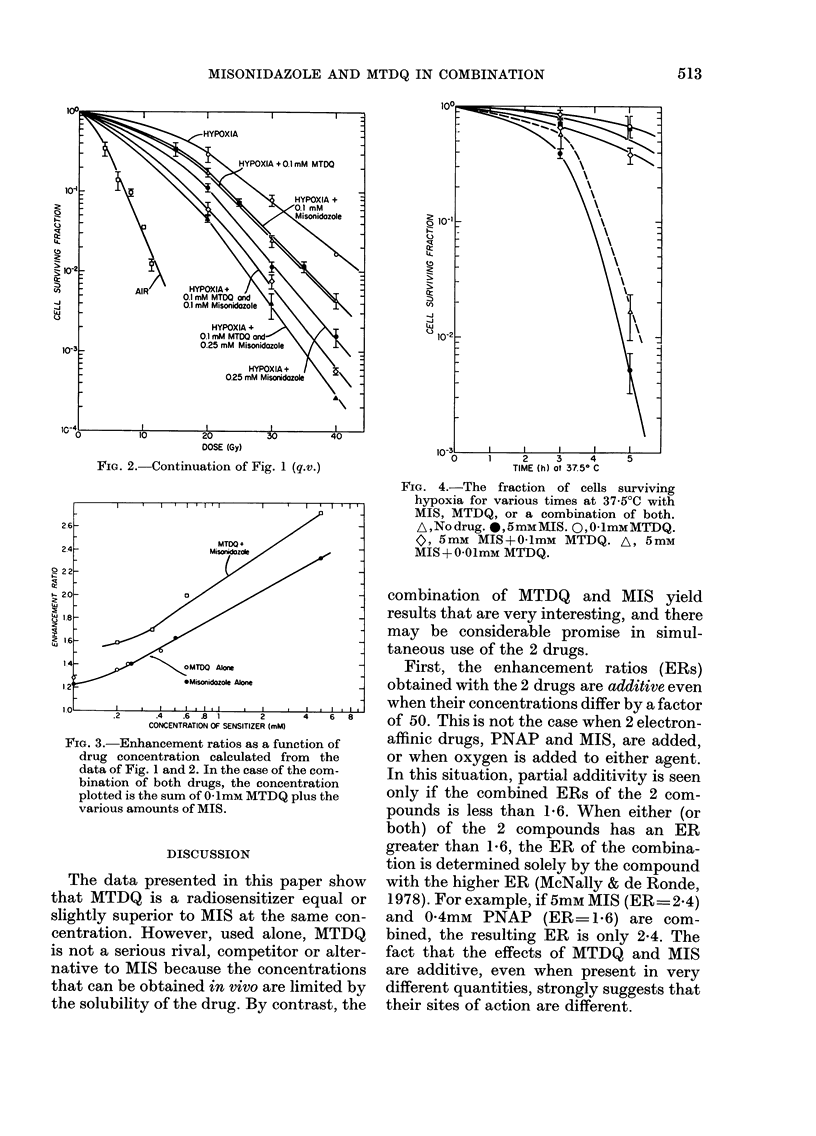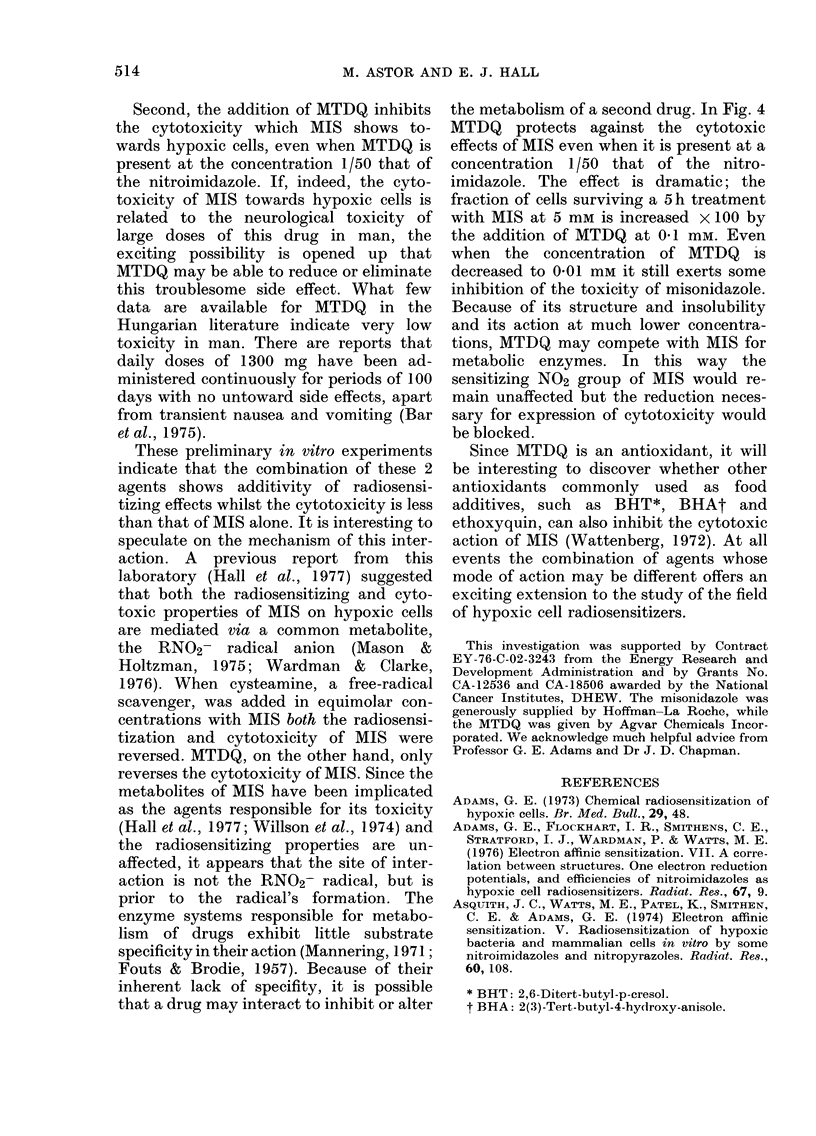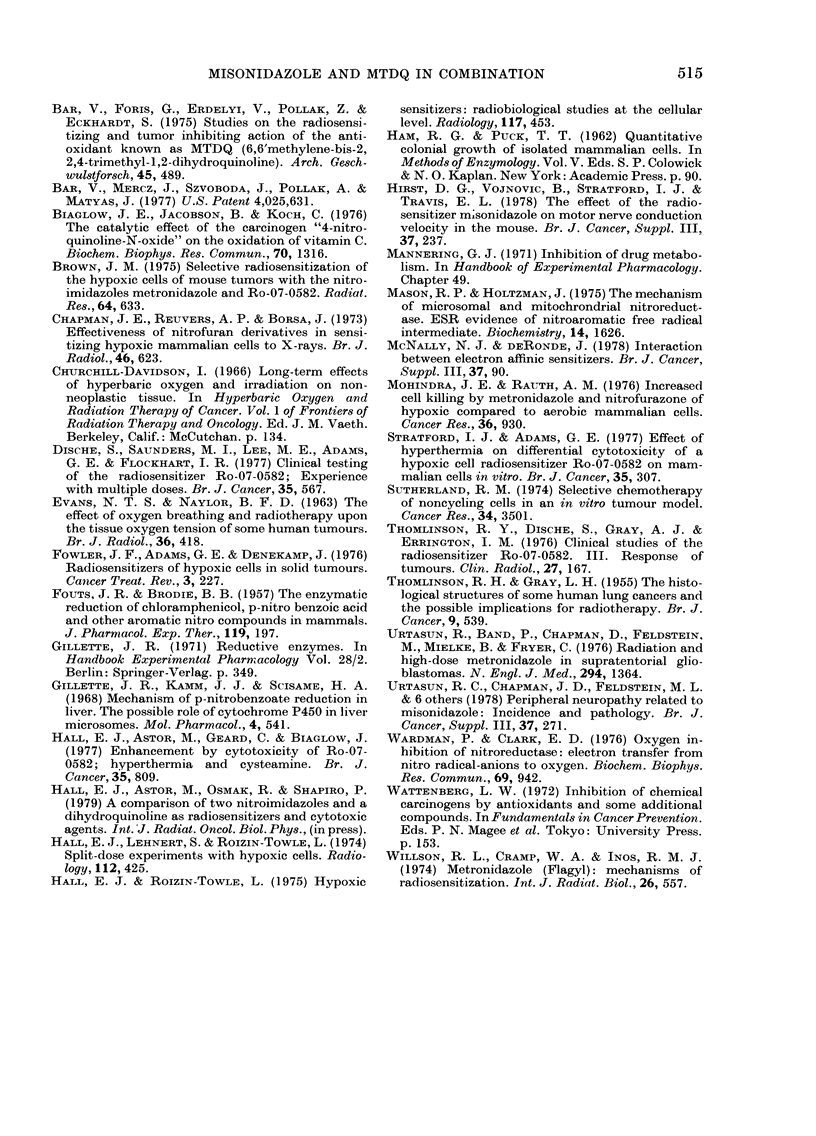# Misonidazole and MTDQ in combination: cytotoxic and radiosensitizing properties in hypoxic mammalian cells.

**DOI:** 10.1038/bjc.1979.94

**Published:** 1979-05

**Authors:** M. Astor, E. J. Hall

## Abstract

A combination of misonidazole and MTDQ (6,6'-methylene-bis-2,2,4 trimethyl-1,2-dihydroquinoline) has been tested for its radiation-sensitizing properties and cytotoxicity, using Chinese hamster V79 cells cultured in vitro. Both compounds sensitize hypoxic cells to the effects of X-rays, and when used in combination their sensitizing properties are additive. By contrast, the presence of MTDQ completely inhibits the cytotoxicity that misonidazole exhibits towards hypoxic cells. These experiments shed some light on the mechanism of action of electron-affinic hypoxic cell sensitizers, and the combination of radiosensitizers suggested may have an application in human cancer radiotherapy by eliminating the neurotoxicity experienced by patients receiving misonidazole during radiotherapy.


*Br. J. Cancer* (1979), **39**, 510

### MISONIDAZOLE AND MTDQ IN COMBINATION: CYTOTOXIC AND RADIOSENSITIZING PROPERTIES IN HYPOXIC MAMMALIAN CELLS

M. ASTOR AND E. J. HALL

*From the Radiological Research Laboratory, Department of Radiology, and the Cancer Center/ Institute of Cancer Research, Columbia University College of Physicians and Surgeons, New York, N.Y., U.S.A.*

Received 4 January 1979 Accepted 7 February 1979

**Summary.—A combination of misonidazole and MTDQ (6,6′-methylene-bis-2,2,4 trimethyl-1,2-dihydroquinoline) has been tested for its radiation-sensitizing properties and cytotoxicity, using Chinese hamster V79 cells cultured *in vitro*.**

**Both compounds sensitize hypoxic cells to the effects of X-rays, and when used in combination their sensitizing properties are additive. By contrast, the presence of MTDQ completely inhibits the cytotoxicity that misonidazole exhibits towards hypoxic cells.**

**These experiments shed some light on the mechanism of action of electron-affinic hypoxic cell sensitizers, and the combination of radiosensitizers suggested may have an application in human cancer radiotherapy by eliminating the neurotoxicity experienced by patients receiving misonidazole during radiotherapy.**

MISONIDAZOLE (MIS) is an electron-affinic compound that selectively sensitizes mammalian cells to the lethal effects of X- and $\gamma$-rays, and is already in use in clinical trials as an adjunct to radiotherapy (Asquith *et al.*, 1974; Brown, 1975; Fowler *et al.*, 1976). MTDQ (6,6′-methylene-bis-2,2,4 trimethyl-1, 2-dihydroquinoline) is an antioxidant that was initially developed as a food additive, but has recently been the subject of preliminary clinical investigations in Hungary and limited *in vitro* experimentation in the United States (Bar *et al.*, 1975, 1977; Hall *et al.*, 1978).

It has been shown in numerous biological systems that hypoxic cells are relatively resistant to killing by sparsely ionizing radiations such as X- or $\gamma$-rays. It is not known with certainty whether human tumours contain viable hypoxic cells that limit their curability by ionizing radiations, but from histological evidence and by analogy with animal tumours it is likely that they do (Thomlinson & Gray, 1955; Evans & Naylor, 1963). Various methods have been proposed to counter the problem of hypoxic cells. These include the use of high-pressure oxygen chambers, hyperthermia, radiations with a high linear-energy-transfer and, most recently, the introduction of chemical radiosensitizers that interact with radiation to increase specifically the sensitivity of hypoxic cells while not affecting the response of normal cells (Fowler *et al.*, 1976; Churchill-Davidson, 1966). A number of compounds have been tested as radiosensitizers *in vitro* and *in vivo*. Chapman *et al.* (1973) linked the radiosensitizing properties to the nitro group, whilst Adams and his colleagues defined the properties necessary for a compound to be used clinically, and established the relationship between electron affinity and radiosensitizing efficiency (Adams, 1973; Adams *et al.*, 1976). As a result of this diverse experimentation, the nitroimidazoles have been shown to possess the necessary properties and have emerged as leading candidates for clinical use. Currently, 2 of these compounds, misonidazole and metronidazole, are undergoing clinical trials in a number of different countries (Urtasun *et al.*, 1976; Thomlinson *et al.*, 1976; Dische *et al.*, 1977).

In addition to preferentially radiosensitizing hypoxic cells, the nitroimidazoles also exhibit a temperature-dependent cytotoxicity towards cells deficient in oxygen (Hall & Roizin-Towle, 1975; Mohindra & Rauth 1976; Stratford & Adams, 1977; Sutherland, 1974; Hall *et al.*, 1977). Generally speaking, 2-nitroimidazoles have been demonstrated to be more cytotoxic than the corresponding 5-nitroimidazoles. It has been postulated that the cytotoxicity is due to intermediate metabolites of the nitroreductase enzyme systems which only function in the absence of oxygen. These enzymes catalyse the reduction of the nitro group to the amino group. Two of the known metabolites, namely the nitroso and hydroxylamine intermediates, are known carcinogens and cytotoxic agents (Gillette, 1971; Gillette *et al.*, 1968; Biaglow *et al.*, 1976; Hall *et al.*, 1977; Willson *et al.*, 1974).

The clinical use of MIS in radiotherapy patients has been limited because of the occurrence of peripheral neuropathy and symptoms arising from central-nervous-system damage when large doses are given repeatedly. It is thought that the peripheral neuropathy is a consequence of damage to the myelin sheath surrounding the nervous fibres (Urtasun *et al.*, 1978; Hirst *et al.*, 1978; Dische *et al.*, 1977). It has not been established whether there is a correlation between the neurological toxicity found in patients and the cytotoxicity towards hypoxic cells that has been shown with cell cultures *in vitro*.

Recent experiments have shown that MTDQ is a radiosensitizer with an effectiveness very similar to that of MIS (Hall *et al.*, 1979). Its use is limited because relatively low concentrations only can be obtained in the human. However, it shows a very low toxicity compared with MIS and it may be of interest for this reason (Bar *et al.*, 1975). The present communication describes experiments involving the simultaneous use of MIS and MTDQ in order to determine the sensitizing properties and the cytotoxicity of this combination of 2 widely different agents.

### MATERIALS AND METHODS

Standard culture techniques were used to grow Chinese hamster V79 cells in GIBCO F-10 culture medium, supplemented with 10% foetal calf serum, and antibiotics (Ham & Puck, 1962).

For radiation or cytotoxicity experiments under hypoxia, cells were treated in suspension in glass ampoules. To induce hypoxia, a large number of cells was crowded into a small volume of medium so that $O_2$ was reduced to a low level by cell metabolism and respiration. This method has been widely used, and described in detail elsewhere (Hall *et al.*, 1974). The essential steps are as follows: cells from a number of actively growing, partially confluent, stock flasks were harvested by trypsinization, washed to remove excess trypsin, and the concentration of cells determined by counting with a Coulter electronic cell counter. This cell suspension was then divided into several parts and the cell concentration in each adjusted to $2\times10^6$ cells/ml, adding one or another drug at an appropriate concentration according to the plan of the particular experiment. MIS dissolved readily in the cell-culture medium, but in the case of MTDQ, the drug had first to be dissolved in a solvent such as Tween-80. This solution was then diluted with medium until the concentration of the Tween-80 represented no more than 0·1% of the culture medium; this was necessary because the solvent *per se* was found to be cytotoxic. From each of the cell suspensions, containing various concentrations of one drug, 2 drugs, or no drug at all, series of glass ampoules were filled by pipetting 1 ml of the cell suspension into each. These ampoules were then handled in the following way in order to render the cells hypoxic. The ampoules were flushed with high-purity $N_2$ plus 5% $CO_2$ in order to displace the air above the cell suspension. Each ampoule was heat-sealed before it was transferred to a water bath at 37·5°C, where it was continuously shaken and tumbled end-to-end to keep the cells in suspension. This procedure was maintained for 1 h to allow the cells to scavenge the $O_2$ dissolved in the medium by normal respiration and metabolism. Proof that this system produces adequate levels of hypoxia is evidenced by an oxygen enhancement ratio (OER) in excess of 3 when aerated and hypoxic cells are exposed to acute doses of $^{60}$Cobalt $\gamma$-rays. A parallel series of ampoules was filled with cells at a concentration of $10^4$/ml; these were gassed with a mixture of air and 5% $CO_2$ before being heat-sealed. Because of the lower number of cells, these ampoules remained aerated throughout. For the periods of time used in these experiments, the plating efficiency for both groups of cells, aerated and hypoxic, remained high (typically 70%) and unaffected by the experimental conditions used.

Following this procedure the cells were irradiated with $\gamma$-rays, or subjected to various periods in the water bath at 37·5°C to assess cytotoxicity. The source of $\gamma$-rays was a $^{60}$Cobalt teletherapy unit, directed vertically upwards so that the cells were irradiated through the bottom of the glass ampoules. At a treatment distance of 40 cm the dose rate was computed to be 2·05 Gy/min. Heat treatments were carried out by immersing the ampoules in a water bath at 37·5°C controlled to $\pm$0·1°C; the ampoules were continuously tumbled and shaken during this heat treatment to prevent attachment of the cells to the glass surface.

At the conclusion of all treatments, each ampoule was agitated on a vortex mixer to resuspend the cells, after which it was opened and various aliquots of the cell suspension plated into Falcon tissue-culture flasks containing fresh growth medium. After incubation for 8 days at 37·5°C, cells were fixed and stained, and the number of macroscopic colonies counted by a projection technique.

FIG. 1.—Survival data for Chinese hamster V79 cells exposed to graded doses of $^{60}$Co $\gamma$-rays under aerated or hypoxic conditions in the presence or absence of various concentrations of misonidazole (MIS), MTDQ, or both in combination. Standard errors are shown where larger than the points plotted. The curves were fitted by eye.

### RESULTS

Fig. 1 and 2 show the results of experiments in which Chinese hamster V79 cells were exposed to graded doses of $^{60}$Cobalt $\gamma$-rays under aerated and hypoxic conditions, in the presence of various concentrations of MIS or MTDQ or a combination of both. In Fig. 3, the enhancement ratio is plotted as a function of drug concentration for MTDQ alone, or both in combination. The enhancement ratio is defined as the ratio of doses in the absence and presence of the drug required to produce the same biological effect; the values plotted in Fig. 3 were taken from the experimental data in Figs. 1 and 2.

Fig. 4 shows the result of an experiment in which Chinese hamster V79 cells were exposed for various periods of time at 37·5°C to MTDQ alone, MIS alone, or a combination of both. It is evident from the figure that 0·1 mM of MTDQ does not show significant cytotoxicity, whilst the addition of this quantity of the antioxidant completely blocks the substantial cytotoxicity produced by 5 mM of MIS. The data in Fig. 4 are from one large self-contained experiment, which has however been repeated 5 times with essentially similar results.

FIG. 2.—Continuation of Fig. 1 (*q.v.*)

FIG. 3.—Enhancement ratios as a function of drug concentration calculated from the data of Fig. 1 and 2. In the case of the combination of both drugs, the concentration plotted is the sum of 0·1mM MTDQ plus the various amounts of MIS.

FIG. 4.—The fraction of cells surviving hypoxia for various times at 37·5°C with MIS, MTDQ, or a combination of both. △, No drug. ●, 5mM MIS. ○, 0·1mM MTDQ. ◇, 5mM MIS+0·1mM MTDQ. △, 5mM MIS+0·01mM MTDQ.

DISCUSSION

The data presented in this paper show that MTDQ is a radiosensitizer equal or slightly superior to MIS at the same concentration. However, used alone, MTDQ is not a serious rival, competitor or alternative to MIS because the concentrations that can be obtained *in vivo* are limited by the solubility of the drug. By contrast, the combination of MTDQ and MIS yield results that are very interesting, and there may be considerable promise in simultaneous use of the 2 drugs.

First, the enhancement ratios (ERs) obtained with the 2 drugs are *additive* even when their concentrations differ by a factor of 50. This is not the case when 2 electron-affinic drugs, PNAP and MIS, are added, or when oxygen is added to either agent. In this situation, partial additivity is seen only if the combined ERs of the 2 compounds is less than 1·6. When either (or both) of the 2 compounds has an ER greater than 1·6, the ER of the combination is determined solely by the compound with the higher ER (McNally & de Ronde, 1978). For example, if 5mM MIS (ER=2·4) and 0·4mM PNAP (ER=1·6) are combined, the resulting ER is only 2·4. The fact that the effects of MTDQ and MIS are additive, even when present in very different quantities, strongly suggests that their sites of action are different.

Second, the addition of MTDQ inhibits the cytotoxicity which MIS shows towards hypoxic cells, even when MTDQ is present at the concentration 1/50 that of the nitroimidazole. If, indeed, the cytotoxicity of MIS towards hypoxic cells is related to the neurological toxicity of large doses of this drug in man, the exciting possibility is opened up that MTDQ may be able to reduce or eliminate this troublesome side effect. What few data are available for MTDQ in the Hungarian literature indicate very low toxicity in man. There are reports that daily doses of 1300 mg have been administered continuously for periods of 100 days with no untoward side effects, apart from transient nausea and vomiting (Bar *et al.*, 1975).

These preliminary *in vitro* experiments indicate that the combination of these 2 agents shows additivity of radiosensitizing effects whilst the cytotoxicity is less than that of MIS alone. It is interesting to speculate on the mechanism of this interaction. A previous report from this laboratory (Hall *et al.*, 1977) suggested that both the radiosensitizing and cytotoxic properties of MIS on hypoxic cells are mediated *via* a common metabolite, the $RNO_2^-$ radical anion (Mason & Holtzman, 1975; Wardman & Clarke, 1976). When cysteamine, a free-radical scavenger, was added in equimolar concentrations with MIS *both* the radiosensitization and cytotoxicity of MIS were reversed. MTDQ, on the other hand, only reverses the cytotoxicity of MIS. Since the metabolites of MIS have been implicated as the agents responsible for its toxicity (Hall *et al.*, 1977; Willson *et al.*, 1974) and the radiosensitizing properties are unaffected, it appears that the site of interaction is not the $RNO_2^-$ radical, but is prior to the radical's formation. The enzyme systems responsible for metabolism of drugs exhibit little substrate specificity in their action (Mannering, 1971; Fouts & Brodie, 1957). Because of their inherent lack of specifity, it is possible that a drug may interact to inhibit or alter the metabolism of a second drug. In Fig. 4 MTDQ protects against the cytotoxic effects of MIS even when it is present at a concentration 1/50 that of the nitroimidazole. The effect is dramatic; the fraction of cells surviving a 5 h treatment with MIS at 5 mM is increased ×100 by the addition of MTDQ at 0·1 mM. Even when the concentration of MTDQ is decreased to 0·01 mM it still exerts some inhibition of the toxicity of misonidazole. Because of its structure and insolubility and its action at much lower concentrations, MTDQ may compete with MIS for metabolic enzymes. In this way the sensitizing $NO_2$ group of MIS would remain unaffected but the reduction necessary for expression of cytotoxicity would be blocked.

Since MTDQ is an antioxidant, it will be interesting to discover whether other antioxidants commonly used as food additives, such as BHT*, BHA† and ethoxyquin, can also inhibit the cytotoxic action of MIS (Wattenberg, 1972). At all events the combination of agents whose mode of action may be different offers an exciting extension to the study of the field of hypoxic cell radiosensitizers.

This investigation was supported by Contract EY-76-C-02-3243 from the Energy Research and Development Administration and by Grants No. CA-12536 and CA-18506 awarded by the National Cancer Institutes, DHEW. The misonidazole was generously supplied by Hoffman-La Roche, while the MTDQ was given by Agvar Chemicals Incorporated. We acknowledge much helpful advice from Professor G. E. Adams and Dr J. D. Chapman.

### REFERENCES

ADAMS, G. E. (1973) Chemical radiosensitization of hypoxic cells. *Br. Med. Bull.*, **29**, 48.

ADAMS, G. E., FLOCKHART, I. R., SMITHENS, C. E., STRATFORD, I. J., WARDMAN, P. & WATTS, M. E. (1976) Electron affinic sensitization. VII. A correlation between structures. One electron reduction potentials, and efficiencies of nitroimidazoles as hypoxic cell radiosensitizers. *Radiat. Res.*, **67**, 9.

ASQUITH, J. C., WATTS, M. E., PATEL, K., SMITHEN, C. E. & ADAMS, G. E. (1974) Electron affinic sensitization. V. Radiosensitization of hypoxic bacteria and mammalian cells *in vitro* by some nitroimidazoles and nitropyrazoles. *Radiat. Res.*, **60**, 108.

* BHT: 2,6-Ditert-butyl-p-cresol.
† BHA: 2(3)-Tert-butyl-4-hydroxy-anisole.

BAR, V., FORIS, G., ERDELYI, V., POLLAK, Z. & ECKHARDT, S. (1975) Studies on the radiosensitizing and tumor inhibiting action of the antioxidant known as MTDQ (6,6'methylene-bis-2, 2,4-trimethyl-1,2-dihydroquinoline). *Arch. Geschwulstforsch*, **45**, 489.

BAR, V., MERCZ, J., SZVOBODA, J., POLLAK, A. & MATYAS, J. (1977) *U.S. Patent* 4,025,631.

BIAGLOW, J. E., JACOBSON, B. & KOCH, C. (1976) The catalytic effect of the carcinogen "4-nitroquinoline-N-oxide" on the oxidation of vitamin C. *Biochem. Biophys. Res. Commun.*, **70**, 1316.

BROWN, J. M. (1975) Selective radiosensitization of the hypoxic cells of mouse tumors with the nitroimidazoles metronidazole and Ro-07-0582. *Radiat. Res.*, **64**, 633.

CHAPMAN, J. E., REUVERS, A. P. & BORSA, J. (1973) Effectiveness of nitrofuran derivatives in sensitizing hypoxic mammalian cells to X-rays. *Br. J. Radiol.*, **46**, 623.

CHURCHILL-DAVIDSON, I. (1966) Long-term effects of hyperbaric oxygen and irradiation on non-neoplastic tissue. In *Hyperbaric Oxygen and Radiation Therapy of Cancer. Vol.* 1 *of Frontiers of Radiation Therapy and Oncology.* Ed. J. M. Vaeth. Berkeley, Calif.: McCutchan. p. 134.

DISCHE, S., SAUNDERS, M. I., LEE, M. E., ADAMS, G. E. & FLOCKHART, I. R. (1977) Clinical testing of the radiosensitizer Ro-07-0582; Experience with multiple doses. *Br. J. Cancer*, **35**, 567.

EVANS, N. T. S. & NAYLOR, B. F. D. (1963) The effect of oxygen breathing and radiotherapy upon the tissue oxygen tension of some human tumours. *Br. J. Radiol.*, **36**, 418.

FOWLER, J. F., ADAMS, G. E. & DENEKAMP, J. (1976) Radiosensitizers of hypoxic cells in solid tumours. *Cancer Treat. Rev.*, **3**, 227.

FOUTS, J. R. & BRODIE, B. B. (1957) The enzymatic reduction of chloramphenicol, p-nitro benzoic acid and other aromatic nitro compounds in mammals. *J. Pharmacol. Exp. Ther.*, **119**, 197.

GILLETTE, J. R. (1971) Reductive enzymes. In *Handbook Experimental Pharmacology* Vol. 28/2. Berlin: Springer-Verlag. p. 349.

GILLETTE, J. R., KAMM, J. J. & SCISAME, H. A. (1968) Mechanism of p-nitrobenzoate reduction in liver. The possible role of cytochrome P450 in liver microsomes. *Mol. Pharmacol.*, **4**, 541.

HALL, E. J., ASTOR, M., GEARD, C. & BIAGLOW, J. (1977) Enhancement by cytotoxicity of Ro-07-0582; hyperthermia and cysteamine. *Br. J. Cancer*, **35**, 809.

HALL, E. J., ASTOR, M., OSMAK, R. & SHAPIRO, P. (1979) A comparison of two nitroimidazoles and a dihydroquinoline as radiosensitizers and cytotoxic agents. *Int. J. Radiat. Oncol. Biol. Phys.*, (in press).

HALL, E. J., LEHNERT, S. & ROIZIN-TOWLE, L. (1974) Split-dose experiments with hypoxic cells. *Radiology*, **112**, 425.

HALL, E. J. & ROIZIN-TOWLE, L. (1975) Hypoxic sensitizers: radiobiological studies at the cellular level. *Radiology*, **117**, 453.

HAM, R. G. & PUCK, T. T. (1962) Quantitative colonial growth of isolated mammalian cells. In *Methods of Enzymology*. Vol. V. Eds. S. P. Colowick & N. O. Kaplan. New York: Academic Press. p. 90.

HIRST, D. G., VOJNOVIC, B., STRATFORD, I. J. & TRAVIS, E. L. (1978) The effect of the radiosensitizer misonidazole on motor nerve conduction velocity in the mouse. *Br. J. Cancer, Suppl.* III, **37**, 237.

MANNERING, G. J. (1971) Inhibition of drug metabolism. In *Handbook of Experimental Pharmacology.* Chapter 49.

MASON, R. P. & HOLTZMAN, J. (1975) The mechanism of microsomal and mitochrondrial nitroreductase. ESR evidence of nitroaromatic free radical intermediate. *Biochemistry*, **14**, 1626.

McNALLY, N. J. & DERONDE, J. (1978) Interaction between electron affinic sensitizers. *Br. J. Cancer, Suppl.* III, **37**, 90.

MOHINDRA, J. E. & RAUTH, A. M. (1976) Increased cell killing by metronidazole and nitrofurazone of hypoxic compared to aerobic mammalian cells. *Cancer Res.*, **36**, 930.

STRATFORD, I. J. & ADAMS, G. E. (1977) Effect of hyperthermia on differential cytotoxicity of a hypoxic cell radiosensitizer Ro-07-0582 on mammalian cells *in vitro*. *Br. J. Cancer*, **35**, 307.

SUTHERLAND, R. M. (1974) Selective chemotherapy of noncycling cells in an *in vitro* tumour model. *Cancer Res.*, **34**, 3501.

THOMLINSON, R. Y., DISCHE, S., GRAY, A. J. & ERRINGTON, I. M. (1976) Clinical studies of the radiosensitizer Ro-07-0582. III. Response of tumours. *Clin. Radiol.*, **27**, 167.

THOMLINSON, R. H. & GRAY, L. H. (1955) The histological structures of some human lung cancers and the possible implications for radiotherapy. *Br. J. Cancer*, **9**, 539.

URTASUN, R., BAND, P., CHAPMAN, D., FELDSTEIN, M., MIELKE, B. & FRYER, C. (1976) Radiation and high-dose metronidazole in supratentorial glioblastomas. *N. Engl. J. Med.*, **294**, 1364.

URTASUN, R. C., CHAPMAN, J. D., FELDSTEIN, M. L. & 6 others (1978) Peripheral neuropathy related to misonidazole: Incidence and pathology. *Br. J. Cancer, Suppl.* III, **37**, 271.

WARDMAN, P. & CLARK, E. D. (1976) Oxygen inhibition of nitroreductase: electron transfer from nitro radical-anions to oxygen. *Biochem. Biophys. Res. Commun.*, **69**, 942.

WATTENBERG, L. W. (1972) Inhibition of chemical carcinogens by antioxidants and some additional compounds. In *Fundamentals in Cancer Prevention.* Eds. P. N. Magee *et al.* Tokyo: University Press. p. 153.

WILLSON, R. L., CRAMP, W. A. & INOS, R. M. J. (1974) Metronidazole (Flagyl): mechanisms of radiosensitization. *Int. J. Radiat. Biol.*, **26**, 557.